# A randomised controlled trial of Heparin versus EthAnol Lock THerapY for the prevention of Catheter Associated infecTion in Haemodialysis patients – the HEALTHY-CATH trial

**DOI:** 10.1186/1471-2369-13-146

**Published:** 2012-11-02

**Authors:** Jennifer K Broom, Rathika Krishnasamy, Carmel M Hawley, E Geoffrey Playford, David W Johnson

**Affiliations:** 1 Department of Infectious Diseases, University of Queensland, Nambour General Hospital, PO Box 547, Nambour, QLD, 4560, Australia; 2 Department of Nephrology, University of Queensland, at Princess Alexandra Hospital, Brisbane, Australia; 3 Infection Management Service, University of Queensland, at Princess Alexandra Hospital, Brisbane, Australia

**Keywords:** Catheter related blood stream infection (CRBSI), Central venous catheter, Ethanol, Lock therapy, Haemodialysis (HD), Prophylaxis

## Abstract

**Background:**

Tunnelled central venous dialysis catheter use is significantly limited by the occurrence of catheter-related infections. This randomised controlled trial assessed the efficacy of a 48 hour 70% ethanol lock vs heparin locks in prolonging the time to the first episode of catheter related blood stream infection (CRBSI).

**Methods:**

Patients undergoing haemodialysis (HD) via a tunnelled catheter were randomised 1:1 to once per week ethanol locks (with two heparin locks between other dialysis sessions) vs thrice per week heparin locks.

**Results:**

Observed catheter days in the heparin (n=24) and ethanol (n=25) groups were 1814 and 3614 respectively. CRBSI occurred at a rate of 0.85 vs. 0.28 per 1000 catheter days in the heparin vs ethanol group by intention to treat analysis (incident rate ratio (IRR) for ethanol vs. heparin 0.17; 95%CI 0.02-1.63; p=0.12). Flow issues requiring catheter removal occurred at a rate of 1.6 vs 1.4 per 1000 catheter days in the heparin and ethanol groups respectively (IRR 0.85; 95% CI 0.20-3.5 p =0.82 (for ethanol vs heparin).

**Conclusions:**

Catheter survival and catheter-related blood stream infection were not significantly different but there was a trend towards a reduced rate of infection in the ethanol group. This study establishes proof of concept and will inform an adequately powered multicentre trial to definitively examine the efficacy and safety of ethanol locks as an alternative to current therapies used in the prevention of catheter-associated blood stream infections in patients dialysing with tunnelled catheters.

**Trial Registration:**

Australian New Zealand Clinical Trials Registry ACTRN12609000493246

## Background

Catheter associated infection is a difficult clinical problem in renal medicine with blood stream infections occurring in up to 40% of patients with haemodialysis catheters, conferring significant rates of morbidity and mortality [1,2]. Methods to reduce rates of infection include intraluminal and extraluminal approaches. Extraluminal methods include topical antimicrobial dressings at the catheter exit site [3], and tunnelling of the catheter [4]. Catheter lock solutions and coating catheters with antimicrobial substances [5] address intraluminal sources of infection. Prophylactic lock solutions that have been studied include antibiotic locks and sodium citrate locks [6,7]. Consensus within the literature about the efficacy of different lock solutions is limited because the methodology of trials reported is often poor, with frequent use of historical controls, or reports in the form of case series with no control group. In addition, there is variation between trials in the concentrations, and length of dwell time with each type of lock study, and these variations in combination with often disparate patient groups and diverse catheter types make conclusions from the limited literature available difficult to reach. Concerns around the use of antibiotic lock solutions include the potential for the development of bacterial resistance [8], limited efficacy, and toxicity from blood stream levels of lock antibiotics, such as gentamicin [9]. Moreover, sodium citrate locks were shown in a recent randomised controlled trial to provide no significant protection against catheter-associated infection and to increase the risk of catheter thrombosis [6]. Ethanol is attractive as a prophylactic lock solution as it is bactericidal by protein denaturation, has a broad range of antimicrobial activity [10], is relatively inexpensive, is metabolised by humans, and does not damage catheters by prolonged exposure [11,12].

To date, there have only been a small number of studies examining the clinical utility of prophylactic ethanol lock therapy, although these have been limited by small numbers, short-follow-up durations, absence of controls or use of historical controls, failure to evaluate dialysis patients and conflicting findings [13-18].

The aim of the present study was to prospectively determine whether a 48 hour ethanol lock was a practical and effective strategy for reducing catheter-associated blood stream infections and prolonging the life of the tunnelled catheter in HD patients. Ethanol lock-related complications were also evaluated.

## Methods

This study protocol has been published elsewhere [19]. A prospective open-label randomised controlled trial design was used to allocate adult patients dialysing through a tunnelled central venous catheter 1:1 to receive 3mL of intravenous grade 70% ethanol into each lumen of the catheter once per week and standard heparin locks for other dialysis days, or to receive heparin locks only. The primary outcome measure was time to the first episode of CRBSI. Secondary outcomes included incidence of CRBSI caused by different pathogens, time to exit site infection, time to infection-related catheter removal, time to catheter removal and adverse reactions.

**Figure 1:**
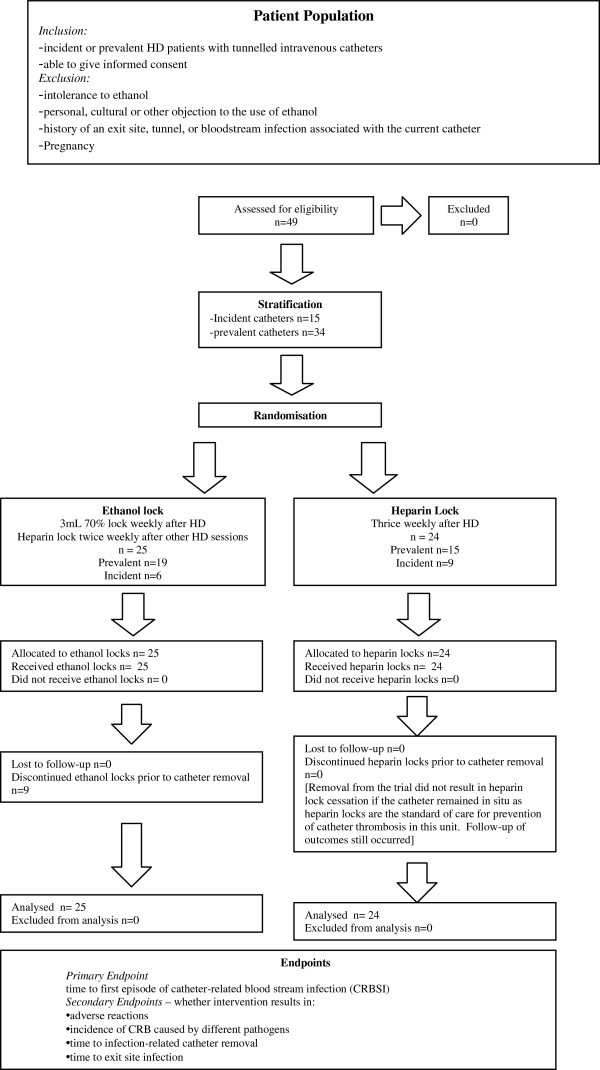
Patient recruitment, stratification, randomisation and analysis in the HEALTHYCATH Trial is represented in a flow diagram.

A flow diagram demonstrating patient recruitment and flow throughout the trial is shown (Figure 1). Ethical approval for the trial was obtained from the Princess Alexandra Human Research Ethics Committee. Participants were selected from patients undergoing HD at the Princess Alexandra Hospital, Logan Hospital, and Redland Hospital in Brisbane, Queensland, with prevalent and incident tunnelled catheters in the internal jugular vein. Inclusion criteria were; adults > 18 years, the presence of a tunnelled intravenous catheter and the ability to give informed consent. Exclusion criteria included pregnancy or breast feeding, religious or personal objection to the use of ethanol, intolerance of ethanol, and a history of an exit site, tunnel or blood stream infection associated with the current catheter.

The study was a prospective, open-label, randomised, controlled trial. Both incident catheters (recruitment for the trial at catheter insertion) and prevalent catheters (recruited to the trial with a previously inserted catheter) were entered into the trial. Incident and prevalent catheters were stratified prior to randomisation. The trial design was not blinded as the odour of ethanol can be discerned by patients and staff during administration which would compromise the blinding. Adequate allocation concealment was ensured using a centralised computer generated block randomisation procedure. Randomisation occurred on the day that trial consent was obtained and was conducted by calling a centralised number to allocate the patient to a trial arm. Participants were randomly allocated to receive either thrice weekly standard heparin locks (Heparin sodium 5000 U/mL, Hospira, Germany) (control group) or weekly catheter instillation of 3 mL intravenous-grade 70% ethanol for 48 hours together with standard heparin locks (ethanol group) following the remaining 2 HD sessions each week. Ethanol 99% (dehydrated alcohol USP injection, Phebra Pty Ltd, NSW 2066, Australia) was diluted to 70% with sterile water for injections BP (AstraZenica Pty Ltd, 2–6 Alma Road, North Ryde, NSW, 2113) with the addition of 7mL ethanol to 3mL water (total 10mL, 3 mL of which was instilled in each lumen) in a syringe by the nurses on the haemodialysis unit. The locking agents were instilled into each lumen of the catheter at the end of a HD session and left in situ until the next dialysis session when they were aspirated. Ethanol locks were not administered more frequently than once per week due to concerns about the risk of catheter thrombosis caused by a reduction in heparin lock dwell time.

Patients in each trial arm received catheter care as per the local haemodialysis unit protocol which included the following; cleansing the catheter site and changing of dressing at each use: existing dressing removed and alcoholic chlorhexidine 70% used to cleanse site; Medihoney ^TM^ Antibacterial Wound Gel ^TM^ applied around catheter exit site ; new Tegaderm ^TM^ I.V. Transparent Dressing applied. Patients with suspected CRBSI (assessed clinically as the presence of fever or other signs of sepsis in a patient with an indwelling central venous catheter, with no alternative source of infection identified) were investigated with blood cultures drawn from peripheral veins and from catheter lumens, removal of the catheter with tip culture, and empirical systemic antibiotic administration: Dicloxacillin 1 gram or Cefazolin 1 gram (if non-severe penicillin allergy) or Vancomycin 1 gram (if severe penicillin allergy or if known MRSA carrier) and Gentamicin 2–3 mg/kg. The primary outcome measure was time to the first episode of CRBSI. Infections related to catheters were defined as per Kidney Disease Outcomes Initiative (KDOQI) definitions (Vascular Access 2006 Work Group. Clinical practice guidelines for vascular access. Am J Kidney Dis 2006;48(Suppl 1): S176–S247). Exit-site infection was defined as inflammation confined to the area surrounding the catheter exit site, not extending superiorly beyond the cuff, with excudate culture confirmed to be positive. Tunnel infection was defined as pain and inflammation of the tunnel superior to the cuff which may also have drainage of pus from the exit site that was culture positive. Catheter-related blood stream infection (CRBSI) was defined as positive blood cultures for the presence of bacteria with or without accompanying fever. Definite CRBSI required the same organism from a semiquantitative culture of the catheter tip (>15 colony-forming units per catheter segment) and from a peripheral or catheter blood sample in a symptomatic patient with no other apparent source of infection. Probable CRBSI required defervescence of symptoms after antibiotic therapy in the setting in which blood cultures confirmed infection, but catheter tip did not in a patient with no other apparent source of infection. Possible CRBSI required defervescence of symptoms after antibiotic treatment or after removal of catheter in the absence of laboratory confirmation of bloodstream infection in a symptomatic patient with no other apparent source of infection. Secondary outcomes included incidence of CRBSI caused by different pathogens, time to exit site infection, time to infection-related catheter removal, time to catheter removal and adverse reactions. Flow problems were defined as catheter blockage or reduced flow rates that required catheter removal (failing to respond to thrombolytic therapy) or precipitated removal from the trial. Mechanical dysfunction was any dysfunction of the catheter such as a split, but not including catheter blockage, which required catheter removal. Patients were followed until the removal of the central venous catheter.

### Statistical analysis

Results were expressed as number (%) for categorical data, mean ± standard deviation for continuous normally distributed data and median [interquartile range] for continuous data that was not normally distributed. Count data were described as the number of events observed, the mean/1000 catheter days and the 95% confidence intervals for the estimation of the mean. Kaplan-Meier plots were used to analyse catheter survival and the log rank test was used to examine the statistical differences in the survival curves. The incidence of adverse events was compared between the 2 groups using negative binomial regression. Results of negative binomial regression analyses were reported as incidence rate ratio (IRR) and 95% confidence interval (CI) with a p value testing the null hypothesis: IRR=1. Prospective power calculations indicated that the study would have adequate statistical power (80% probability) to detect a clinically significant increase in infection-free survival from 200 days to 400 days if 56 patients were recruited into each arm (total 112 patients). These assumptions were based on local data in HD patients at the Princess Alexandra Hospital. A recruitment period of 3 years was anticipated based on rates of tunnelled central venous catheter dialysis at the initiation of the study. All analyses were carried out using Graphpad Prism version 5 software and Stata SE version 11.0. All data were analysed on an intention-to-treat basis. P values < 0.05 were considered significant.

## Results

### Participants

Of a planned 112 participants, 49 patients were recruited from October 2006 to November 2010 (Figure 1). Access to fistula formation and peritoneal catheter insertion at the Princess Alexandra Hospital improved dramatically during the study period, thereby markedly reducing the rate of recruitment to the study. During the study, annual PD Tenckhoff catheter insertion rates increased by approximately 40%, whilst prevalent HD catheter rates fell from in excess of 30% to 3% by the end of the study. A decision was therefore made to close the trial before the planned recruitment had been completed. Of 49 patients, 25 were randomised to the ethanol lock arm of the trial and 24 to the heparin lock arm. There were no significant differences in age, gender, or aetiology of chronic kidney disease between the two groups (Table 1).

**Table 1:** Patient characteristics in ethanol lock and heparin lock arms are shown

**Variable**	**Ethanol lock (n=25)**	**Heparin lock (n=24)**
Gender		
-→Male	13 (52%)	11 (46%)
-→Female	12 (48%)	13 (54%)
Age (years, mean±SD)	52±18	64±16
Duration of current catheter use prior to trial entry (median days)	25	18.5
Aetiology of chronic kidney disease		
-→Diabetic nephropathy	8 (32%)	6 (25%)
-→Glomerulonephritis	8 (32%)	7 (29%)
-→Polycystic kidney disease	2 (8%)	2 (8%)
-→Hypertension	1 (4%)	3 (13%)
-→Chronic interstitial nephritis	1 (4%)	1 (4%)
-→Analgesic nephropathy	1 (4%)	1 (4%)
-→Other	4 (16%)	4 (16%)

#### Catheter survival

**Figure 2:**
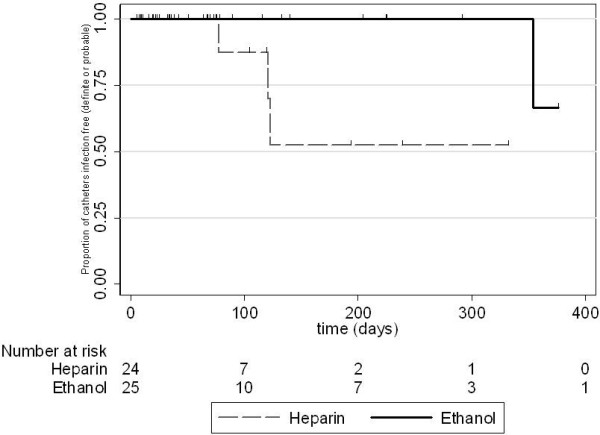
**Kaplan-Meier infection-free survival curves for the heparin and ethanol groups.** Censoring events are indicated in the graph. The numbers at risk at each time point are shown below the abscissa.

A total of 3614 catheter days were observed in the ethanol lock arm and 1834 catheter days in the heparin lock arm from trial entry (Table 2). The intended primary outcome analysis, time to catheter association blood stream infection, definite or probable is shown in Figure 1. There were only 3 events in the heparin group and 1 event in the ethanol group for this outcome. Applying tests to assess differences in time to event was not appropriate with the numbers observed. Numerous censoring events occurred (Figure 2); the main reason being that the catheters were no longer required as permanent access was able to be accessed.

**Table 2:** Outcomes of the trial for ethanol lock and heparin lock arms are shown

**Variable**	**Ethanol lock**	**Heparin lock**	**IRR (95% CI ); p value**
	**(/1000 catheter days; 95%CI)**	**(/1000 catheter days; 95%CI)**	
Total observation period from trial entry (days)	3614	1834	
**Total Complications (intention to treat): infective and non-infective**	9 (2.5; 95%	8 (4.4; 95%	0.57 (0.22-1.5);
	CI 1.1-4.7)	CI 1.9-8.6)	p = 0.25
***Non-infective***	7 (1.9; 95%	4 (2.2; 95%	0.88 (0.26- 3.0);
	CI 0.78-4.0)	CI 0.59-5.6)	p=0.85
-→Flow difficulties	5 (1.4; 95%	3 (1.6; 95%	0.85 (0.20-3.5);
	CI 0.45-3.2)	CI 0.32-4.8)	p=0.82
-→Thrombosis	0	0	
-→Mechanical dysfunction	2	1	
***Infective***	2* (0.55; 95%	4 (2.2; 95%	0.25 (0.05-1.4);
	CI 0.067-2.0)	CI 0.59-5.6)	p=0.113
-→CA-BSI - definite or probable^¥^	1 (0.28; 95%	3 (0.85; 95%	0.17 (0.02-1.63);
	CI.00-1.5)	CI 0.20-3.5)	p=0.12
-→CA-BSI suspected	0	1	
-→Exit site infection	1	0	
-→Tunnel infection	0	0	

* This number represents all infectious complications on an intention-to-treat basis. One CA-BSI occurred in a catheter after exit from the trial in the ethanol arm. Therefore, in Table 4, the infectious complications resulting in trial exit are listed as 1 because the second infection occurred after trial exit.

#### Catheter complications (non infective)

Catheter complications resulting in removal of the catheter occurred in the ethanol vs heparin groups at rates of 1.9 (95% CI 0.78 - 4.0) vs 2.2 (95% CI 0.59 - 5.6) events per 1000 catheter days. Comparing these rates, the incident rate ratio (IRR) for ethanol compared with heparin was 0.88 (95% CI 0.26 – 3.0; p = 0.85. Of these complications, flow difficulties were the most frequent event observed. The rates were 1.4 (95% CI 0.45-3.2) vs 1.6 (95% CI 0.32-4.8) per 1,000 catheter days for ethanol vs heparin groups respectively, IRR 0.85 (95% CI 0.20-3.5; p =0.82).

#### Catheter-related infections

Catheter-related blood stream infections (defined as probable and definite CR-BSIs) occurred on intention to treat analysis in ethanol vs heparin arms at rates of 0.28 (95% CI 0.00-1.5) vs 0.85 (95% CI 0.20-3.5) per 1000 catheter days (IRR 0.17; 0.02-1.63; p=0.12: Table 2). Comparing the rates of all infective events (included definite or probable CR-BSIs, suspected CI-BSIs and exit site and tunnel infections), the rates in the ethanol vs heparin groups were 0.55 (95% CI 0.07 – 2.0) vs 2.2 (95% CI 0.59-5.6) per 1000 catheter days (IRR 0.25; 95% CI −0.05-1.4; p = 0.113).

Causative pathogens are listed (Table 3). No CR-BSI infections occurred in the ethanol group while receiving ethanol locks, although one infection occurred in a patient who had previously received ethanol locks, 303 days after trial exit due to disrupted ethanol supply. In the intention to treat analysis, this infection was included in the ethanol arm. The organisms isolated in the 3 patients on the heparin arm who had CR-BSI were: *Staphylococcus aureus, Enterobacter cloacae,* and *Staphylococcus hominis*. One patient met criteria for definite CR-BSI (patient 2, isolating *Staphylococcus aureus* from both blood cultures and catheter tip culture). The other two patients met criteria for probable CR-BSI. CR-BSIs occurred after 78, 134, and 135 days on the trial. All 3 catheters were removed. One patient in the ethanol arm acquired a definite CR-BSI in which the causative organism was *Staphylococcus aureus*, cultured in blood cultures from both the tunnelled catheter and peripherally and from the catheter tip on removal. This infection occurred 303 days after cessation of ethanol lock therapy. One exit site infection from which methicillin resistant *Staphylococcus aureus* (MRSA) was cultured from an exit site swab occurred in the ethanol arm resulting in removal of the catheter. No exit site infections were documented in the heparin arm. Tunnel infections did not occur in either arm.

**Table 3:** Causative pathogens for catheter-related blood stream infections occurring during the trial

**Code**	**Lock solution**	**Days on trial**	**Organism isolated**	**Isolated from**
2	Heparin	78	*Staphylococcus aureus*	Blood culture 1 set, catheter tip >100 colony forming units (cfu)
24	Heparin	134	*Enterobacter cloacae*	Blood culture - 1 set
27	Heparin	135	*Staphylococcus hominis*	Blood culture - 2 sets, 2 days apart
35	Ethanol	51 (infection occurred 303 days later after trial exit)	*Staphylococcus aureus*	Blood culture - 2 sets
				-→catheter 10hrs,
				-→peripheral 11hrs
				catheter tip >100cfu

#### Exit from trial

Reasons for patients exiting from trial are outlined in in Table 4. Of the non-infectious reasons for removal from the trial, four patients in the ethanol arm were removed from the study at their own request. The first was removed after 126 days on the trial due to a problem aspirating an ethanol lock which required a single flush of the catheter to resolve. The second patient complained of stinging at the catheter exit site on administration of the ethanol lock and was removed from the trial after 2 days. The third patient was removed from the trial at their own request after 8 days with no documentation as to the reason in the patient record. The fourth patient complained of dry lips, being thirsty, and having “flow problems”, although the flow problems were not documented by the clinical staff the patient was removed from the trial after 15 days. No patients in the heparin arm requested removal from the trial.

**Table 4:** Exit from trial and/or end of study events for ethanol lock and heparin lock arms are shown

**Reason for exit from the trial**	**Ethanol Lock**	**Heparin Lock**	**Statistical analysis (where appropriate)**
			**IRR (95% CI ); p value**
**Catheters removed (total)**	25	24	IRR 0.53 (0.30 -0.93)
			P=0.03
**Catheter no longer required**	6	12	IRR 0.25 (0.10-0.68)
			P=0.006
**Infectious complications**	1	4	See Table 2
**Non infectious reasons for exit from trial**	18*	8^#^	

*Split catheter (1), flow problems or blocked catheter (5), intensive care admission unrelated to the trial (1), patient request (4), reduced to twice weekly dialysis (2), not specified (1), relocation to a non trial site (1), non-compliance with trial protocol (1), deceased (1), ethanol supply temporarily ceased (1).

#Catheter fell out (1), flow problems or blocked catheter (3), bleeding from catheter site (1), patient non compliance with dialysis (1), reduced to twice weekly dialysis (1), relocation to non trial site (1).

Five patients in the ethanol arm and three in the heparin arm were removed from the trial because of flow problems. Mechanical problems with the catheter occurred once in both groups. One patient in the ethanol arm had a split catheter, and one patient in the heparin group had a catheter that fell out. Other non-infectious reasons for removal from the trial for patients in the ethanol group were; intensive care admission unrelated to the trial (n=1), reduction to twice weekly dialysis (n=2), relocation to a non-trial site (n=1), non-compliance with trial locks (n=1), patient deceased (n=1), a temporary disruption to ethanol supply (n=1), and one patient that was removed for an unspecified reason. Patients in the heparin group were removed from the trial because of; bleeding from catheter site (n=1), patient non-compliance with dialysis (n=1), reduction to twice weekly dialysis (n-1), and relocation to a non-trial site (n=1).

## Discussion

This is the first study of prophylactic ethanol lock therapy in patients with end-stage kidney failure undergoing HD via a tunnelled central catheter. Although the study did not reach the expected recruitment targets and therefore the results did not reach statistical significance due to a type 2 error, it would appear that ethanol is a safe and potentially effective intervention in this patient group. There was a trend towards increased catheter survival and a decreased rate of catheter-associated blood stream infection with the use of a once per week ethanol lock. These beneficial effects were particularly observed in incident (newly inserted) dialysis catheters.

These findings are in keeping with those of a previous randomised controlled trial of 64 prophylactic treatment periods with a daily ethanol lock or placebo with a dwell time of two hours in 60 haematology inpatients with either tunnelled or untunnelled catheters [17]. Ethanol lock therapy was associated with a significant reduction in catheter-associated blood stream infections in the ethanol arm compared to control.

On the other hand, a second much larger trial of tunnelled catheters in 376 haematology inpatients and outpatients [18] reported that ethanol locks (15 minute dwell time applied daily to inpatients or weekly to outpatients) did not result in a reduction in catheter-associated blood stream infections compared with controls. However, the rates of blood stream infections were low (0.7 vs 1.19 per 1000 catheter days in the ethanol vs placebo groups, p=0.19), such that the study may have been underpowered to show an effect.

An uncontrolled trial of prophylactic ethanol locks in paediatric patients undergoing monoclonal antibody treatment for neuroblastoma was ceased early when 3 of 12 catheters became occluded [14]. The low numbers in this trial and the lack of a control group limit the conclusions that can be drawn, but indicate that ethanol lock treatments should be prospectively studied and appropriately controlled to evaluate this complication in paediatric populations.

A recent study of 90 episodes of 48 hour 60% ethanol locks in 30 HD patients with evaluation of the pre- and post-intervention period (each patient acting as their own control) found a transient increase in catheter dysfunction related to ethanol lock therapy, although no catheter required removal [20]. Each patient had a total of only three consecutive ethanol dwell periods, such that the effect of ethanol locking in the longer term in this patient population was not able to be assessed.

A significant concern in designing this trial was whether thrombotic complications would occur in the ethanol arm as a result of the 48 hour period without a heparin lock *in situ*. Flow problems did occur in both arms with 4 of 25 (16%) ethanol lock patients experiencing flow related problems requiring catheter removal compared to 3 of 24 (13%) heparin lock patients. There was not a significant increase in catheter thrombosis requiring catheter removal in the ethanol lock group. Previous studies of the effects of catheter exposure to ethanol have demonstrated that silicone dialysis catheter integrity is maintained, as assessed by scanning electron microscopy, even after 15 days exposure to 95% ethanol solution [12]. The amount of silicone released was not significantly different when the catheter was submerged in ethanol compared to 0.9% sodium chloride. Mechanical testing of polyurethane and silicone catheters exposed to ten weeks of 70% ethanol proved ongoing structural integrity of both catheter types after prolonged ethanol exposure [11].

Ethanol is an antiseptic agent which is bactericidal by protein denaturation and is active against a wide variety of organisms including Gram-positive bacteria, Gram-negative bacteria, and fungi. *In vitro* studies have been published demonstrating the bactericidal effect of 70% ethanol against plastic-adherent organisms that commonly cause line infections [10]. Complete eradication of Gram-negative bacilli, Gram-positive cocci, and *Candida albicans* biofilms occurred with 30 minutes treatment with 60% ethanol compared to no eradication with 46.7% trisodium citrate. The apparently greater efficacy of ethanol locks with incident catheters in the present study might be potentially related to enhanced effectiveness in catheters in which biofilm has not yet formed. However, the small numbers of incident catheters in the present investigation indicate that further studies are warranted to confirm this finding.

The principal limitations of this study were its open label design, leading to the possibility of reporting bias, and small patient numbers leading to the possibility of type 2 statistical error. Although it was theoretically possible to have blinded this trial by having pharmacy make up syringes filled with heparin or ethanol lock solutions for each patient (as published in a previous prophylaxis trial [17], the distinctive odour of ethanol during catheter instillation would have been discerned by the administering nurse and patient, thereby compromising the integrity of the blinding. Another significant limitation of this study is the large number of patients exiting the trial prior to catheter removal, particularly in the ethanol arm. The specific reasons for trial exit are detailed in the results section. The analysis of the trial has been performed on an intention-to-treat basis consistent with CONSORT guideline recommendations [21]. This methodology does result in a significant number of catheter days in which treatment was not per-protocol on the ethanol arm. The single CR-BSI that occurred in the ethanol lock arm occurred 303 days after cessation of ethanol lock therapy. However it is included in the ethanol lock arm for analysis. Per-protocol analysis would address this issue but would compromise the effect of random allocation and therefore was not used.

Antibiotic lock solutions have been studied in the prevention of catheter associated blood stream infections and do confer a protective effect, as reported in two recent meta-analyses [7,22]. However, the studies were limited with respect to uniformity and long term outcome data, and the use of long term antibiotics in a prophylaxis role still confers the potential for the development of antimicrobial resistance. These studies do support the use of locking solutions, however, and alternative agents such as ethanol could potentially allow for a protective benefit without the risk of resistance.

## Conclusions

In conclusion, although the results of this trial are not statistically significant, the trend towards a longer catheter life and a reduction in catheter associated blood stream infection establishes proof of concept and warrants a multi-centre trial to enroll larger patient numbers to further assess the role of ethanol locks in the prevention of catheter-associated blood stream infections in patients dialysing via tunnelled catheters. Ethanol is an inexpensive, non toxic, bactericidal agent that has potential utility as a lock therapy in preventing catheter associated infections. This trial indicates that the intervention is safe and acceptable to patients undergoing haemodialysis, and may reduce CRBSI. Further multi-centre studies are required to provide power to evaluate the efficacy in prevention of infection. If effective, an agent that does not have an association with the development of antimicrobial resistance would be highly desirable as a prophylaxis agent.

## Competing interests

The authors declare that they have no competing interests.

## Authors’ contributions

JB was the principal investigator; conceived study; participated in design and co-ordination; helped to draft manuscript; read and approved the final manuscript. RK participated in co-ordination; helped to draft manuscript; read and approved the final manuscript. CH participated in co-ordination; helped to draft manuscript; read and approved the final manuscript. GP participated in design and co-ordination; helped to draft manuscript; read and approved the final manuscript. DJ participated in design and co-ordination; helped to draft manuscript; read and approved the final manuscript.

## Pre-publication history

The pre-publication history for this paper can be accessed here:

http://www.biomedcentral.com/1471-2369/13/146/prepub
